# Characteristics of stroke after liver and kidney transplantation

**DOI:** 10.3389/fneur.2023.1123518

**Published:** 2023-03-22

**Authors:** Hanim Kwon, Sung Shin, Chung Hee Baek, Jun Young Chang, Dong-Wha Kang, Sun U. Kwon, Jong S. Kim, Bum Joon Kim

**Affiliations:** ^1^Department of Neurology, Korea University Ansan Hospital, Ansan, Republic of Korea; ^2^Department of Neurology, Asan Medical Center, University of Ulsan College of Medicine, Seoul, Republic of Korea; ^3^Department of Surgery, Asan Medical Center, University of Ulsan College of Medicine, Seoul, Republic of Korea; ^4^Department of Internal Medicine, Asan Medical Center, University of Ulsan College of Medicine, Seoul, Republic of Korea; ^5^Department of Neurology, Gangneung Asan Hospital, University of Ulsan College of Medicine, Gangneung, Republic of Korea

**Keywords:** stroke, etiology, liver transplantation, kidney transplantation, prognosis

## Abstract

**Background:**

The mechanism and characteristics of a post-transplantation stroke may differ between liver (LT) and kidney transplantation (KT), as the associated comorbidities and peri-surgical conditions are different. Herein, we investigated the characteristics and etiologies of stroke occurring after LT and KT.

**Methods:**

Consecutive patients who received LT or KT between January 2005 to December 2020 who were diagnosed with ischemic or hemorrhagic stroke after transplantation were enrolled. Ischemic strokes were further classified according to the etiologies. The characteristics of stroke, including in-hospital stroke, perioperative stroke, stroke etiology, and timing of stroke, were compared between the LT and KT groups.

**Results:**

There were 105 (1.8%) and 58 (1.3%) post-transplantation stroke patients in 5,950 LT and 4,475 KT recipients, respectively. Diabetes, hypertension, and coronary arterial disease were less frequent in the LT than the KT group. In-hospital and perioperative strokes were more common in LT than in the KT group (LT, 57.9%; KT, 39.7%; *p* = 0.03, and LT, 43.9%; KT, 27.6%; *p* = 0.04, respectively). Hemorrhagic strokes were also more common in the LT group (LT, 25.2%; KT, 8.6%; *p* = 0.01). Analysis of ischemic stroke etiology did not reveal significant difference between the two groups; undetermined etiology was the most common, followed by small vessel occlusion and cardioembolism. The 3-month mortality was similar between the two groups (both LT and KT, 10.3%) and was independently associated with in-hospital stroke and elevated C-reactive protein.

**Conclusions:**

In-hospital, perioperative, and hemorrhagic strokes were more common in the LT group than in the KT group. Ischemic stroke subtypes did not differ significantly between the two groups and undetermined etiology was the most common cause of ischemic stroke in both groups. High mortality after stroke was noted in transplantation patients and was associated with in-hospital stroke.

## Introduction

Organ transplantation has become the standard treatment for many end-stage diseases. As increasing number of patients undergo transplantation (1–3) and the rates of early post-transplant survival have improved (3, 4), long-term management of the recipients has become important, with cerebrovascular disease being an important consideration. Although transplantation can reduce the risk of cerebrovascular disease compared to those who did not receive appropriate transplantation (5), still it is a potential cause of death, graft loss, and poor quality of life even in survivors.

Liver transplantation (LT) and kidney transplantation (KT) are the two most frequently performed solid organ transplantations (5–12). Stroke after LT and KT may have different mechanisms and characteristics (5, 7), as the co-morbidities associated with end-stage liver and kidney disease and their peri-surgical conditions differ.

However, previous studies investigating post-transplantation stroke have mainly focused only on its incidence or predictors (5–13). The etiologies and timing of stroke, which are important aspects in understanding and preventing stroke, have been only sparingly covered in this group of patients.

Herein, we investigated the features of post-transplantation stroke and compared the characteristics of stroke occurring after LT and KT. In particular, we explored the mechanisms of stroke to gain a better understanding of stroke in post-transplantation recipients.

## Materials and methods

### Patient selection and grouping

We enrolled consecutive ischemic or hemorrhagic stroke patients who received LT or KT with or without simultaneous pancreas-kidney transplantation (SPKT) between January 2005 and December 2020 at Asan Medical Center, Seoul, Korea. Demographic features, comorbidities, laboratory findings, immunosuppressant use, stroke characteristics including etiologies (subtypes), antithrombotic agents at discharge, and 3-month mortality were obtained from our electronic medical records (EMR). Enrolled patients were dichotomized into two groups, LT and KT, according to their transplantation status. Patients with SPKT were classified into the KT group. The trial was approved by the independent Institutional Review Board (IRB) of Asan medical center, Korea, which waived the need for written consent due to the study's retrospective nature (IRB approval number: 2021-0475).

### Diagnosis and classification of stroke

In our center, the care of transplantation recipients after discharge is performed by the transplantation center in conjunction with a coordinator, whom the patients may contact anytime if any medical condition requiring care occurs. Strokes were reported to the coordinator, and the patients were transferred to our hospital for further management, including post-transplantation care.

In this study, the diagnosis of stroke required both imaging evidence on magnetic resonance imaging (MRI) and the presence of relevant clinical symptoms, whether reported by the patient or observed during a neurologic examination. Acute ischemic lesions were evaluated based on diffusion-weighted imaging (DWI), and white matter changes were investigated using T2-weighted imaging (T2WI) or fluid-attenuated inversion recovery (FLAIR) sequence.

The etiology of ischemic stroke was classified according to the TOAST (Trial of org 10172 in acute stroke treatment) classification; ischemic strokes were classified as large artery atherosclerosis (LAA), small vessel occlusion (SVO), cardioembolism, and other determined and undetermined etiology (14). We diagnosed cardioembolism when a patient had more than moderate risk cardioembolic features and did not satisfy the diagnostic criteria of LAA and SVO. Patients were classified as having stroke of undetermined etiology if they did not have a positive finding to explain the cause of stroke (negative), had more than one possible cause (two or more), or did not complete the minimum evaluation for classification (incomplete).

In-hospital stroke was defined as an ischemic or hemorrhagic stroke occurring in a patient who was initially hospitalized for treatment for another condition (15, 16), including the post-surgical period. In the case of recurrent stroke, only a single earliest event was accounted for by a patient. We defined postoperative 3-month stroke as any ischemic or hemorrhagic infarctions that occurred within 90-days following the transplantation surgery. Among patients with ischemic stroke, the presence of hemorrhagic transformation was assessed only in those who underwent follow-up imaging (either MRI or CT) within 14 days after initial imaging. Multiple lesions and cortical lesion were also evaluated only in the case of ischemic stroke. We defined multiple lesions as discrete non-single hyperintense DWI lesions, regardless of whether they were within the same vascular territory or in different territories. A cortical lesion was defined as an abnormal DWI lesion involving the cortical gray matter of the cerebral or cerebellar hemispheres.

Imaging markers related to the ischemia burden were also investigated. Fazekas score was rated using fluid-attenuated inversion recovery (FLAIR) images, and the score ranged from 0 to 3, reflecting the burden of white matter change (17). In our study, we chose to focus on evaluating deep white matter change to assess the Fazekas score, as it is a more accurate indicator of small vessel disease burden (18) and represents overall white matter changes (19). Cerebral microbleeds were referred to small, rounded, or circular well-defined hypointense lesions within the brain parenchyma with clear margins ranging from 2 to 10 mm on gradient echo (GRE) images (20).

### Statistical analysis

Continuous variables such as age and laboratory findings were analyzed by Mann–Whitney test, and categorical variables, including medical comorbidities, medications, characteristics of stroke, and 3-month mortality, were evaluated by chi-square or Fisher's exact test, as appropriate. Kaplan–Meier curve was used to evaluate the occurrence of stroke events in both groups. We used a two-sided *p*-value of < 0.05 to define statistical significance. Logistic regression analysis was performed to find factors related to mortality at three months, and variables with a *p*-value of same or < 0.05 were included in multivariate analysis. All statistical analyses were performed using SPSS version 26.0 (SPSS, Inc., Chicago, IL).

## Results

### Characteristics of patients with stroke after transplantation

Between January 2005 and December 2020, we evaluated a total of 10,425 patients who underwent transplantation surgery at our hospital, including 5,950 LT recipients and 4,475 KT recipients. During this period, there were 105 (1.8%) and 58 (1.3%) post-transplantation stroke patients in the LT and KT groups including 3 SPKT patients, respectively. Two patients in the LT group who were diagnosed with both ischemic and hemorrhagic stroke simultaneously were included in both groups. The median age was 60.0 (interquartile rage: 54.0–65.5) years, and 73.9% were men.

In the LT group, the most common cause of transplantation was hepatocellular carcinoma (HCC) due to hepatitis virus (*n* = 41, 39.0%). Other causes were comprised of liver cirrhosis due to hepatitis virus (*n* = 28, 26.6%), liver cirrhosis related to alcohol consumption (*n* = 19, 18.1%), HCC related to alcohol consumption (*n* = 6, 5.7%), HCC with unknown cause (*n* = 2, 1.9%), and liver cirrhosis with unknown cause (*n* = 9, 8.6%). In the KT group, diabetes (*n* = 31, 53.4%) was the most frequent cause of transplantation, and ESRD of other causes (*n* = 11, 19.0%), hypertension (*n* = 10, 17.2%), IgA nephropathy (*n* = 4, 6.9%), and polycystic kidney disease (*n* = 2, 3.4%) followed.

Table 1 shows the detailed clinical characteristics of the patients who experienced strokes after LT and KT. the LT group had lower rates of vascular risk factors such as diabetes (35.2 vs. 67.2%, *p* < 0.01), hypertension (45.9 vs. 86.2%, *p* < 0.01), and coronary arterial disease (10.5 vs. 24.1%, *p* = 0.02) than in the KT group. The incidence of active cancer, defined as histologically or cytologically confirmed malignancy and diagnosed or received treatment within 6 months or progressive disease (21), was higher in the LT group (21.0 vs. 5.2%, *p* = 0.01). Laboratory studies at the time of stroke event showed a lower platelet count and a higher international normalized ratio (INR), activated partial thromboplastin time (aPTT), aspartate transaminase (AST) level, and alanine transferase (ALT) levels in the LT group than in the KT group. Conversely, total cholesterol and creatinine levels were higher in the KT group.

**Table 1:** Clinical characteristics of the enrolled patients.

**Characteristics**	**LT group (*n* = 105)**	**KT group (*n* = 58)**	***p*-value**
Age	61.0 (55.0–65.5)	59.0 (49.0–65.0)	0.35
Female sex	27 (25.7)	16 (27.6)	0.80
**Medical history**			
Diabetes mellitus	37 (35.2)	39 (67.2)	< 0.01
Hypertension	52 (49.5)	50 (86.2)	< 0.01
Current smoking	13 (12.4)	4 (6.9)	0.27
Hyperlipidemia	18 (17.1)	12 (30.8)	0.77
Coronary arterial disease	11 (10.5)	14 (24.1)	0.02
Atrial fibrillation	6 (5.7)	7 (12.1)	0.23
Previous stroke	27 (25.7)	23 (39.7)	0.07
Active cancer^*^	22 (21.0)	3 (5.2)	0.01
**Laboratory studies**			
Hemoglobin (g/dL)	9.85 (8.6–12.5)	10.5 (9.4–12.6)	0.29
Platelet (10^3^/μL)	109.5 (56.3–158.0)	180.5 (108.5–240.8)	< 0.01
INR	1.1 (1.0–1.3)	1.0 (1.0–1.2)	< 0.01
aPTT (seconds)	31.1 (27.8–39.4)	28.1 (28.1–32.9)	0.01
C-reactive protein (mg/L)	1.3 (0.2–4.2)	1.0 (0.2–5.6)	0.86
Total cholesterol (mg/dL)	121.5 (89.0–156.0)	152.0 (108.0–181.0)	0.01
Creatinine (mg/dL)	1.1 (0.8–1.5)	1.3 (1.0–2.0)	0.03
BUN (mg/dL)	24.0 (17.5–35.5)	25.0 (16.5–37.0)	0.94
Estimated GFR	65.1 (34.9-86.9)	56.4 (39.8-91.7)	0.97
AST (U/L)	40.0 (22.3–76.3)	22 (15.8–27.3)	< 0.01
ALT (U/L)	39.5 (20.0–85.8)	17.5 (13.0–26.3)	< 0.01
Albumin (g/dL)	3.5 (3.2–3.8)	3.1 (2.6–3.6)	< 0.01
Total bilirubin (mg/dL)	1.3 (0.7–3.5)	0.6 (0.4–0.8)	< 0.01
**Immunosuppressant**			
Steroid	60 (57.1)	51 (87.9)	< 0.01
Cyclosporine	15 (14.3)	18 (69.0)	0.01
Tacrolimus	79 (75.2)	38 (65.5)	0.19
Mycofenolic acid	56 (53.3)	30 (51.7)	0.84
Others	3 (2.9)	9 (15.5)	0.01

Numbers are presented as the median (interquartile range) or number (percentage).

LT, liver transplantation; KT, kidney transplantation; INR, international normalized ratio; aPTT, activated partial thromboplastin time; BUN, blood urea nitrogen; GFR, Glomerular filtration rate; AST, aspartate aminotransferase; AST, aspartate aminotransferase; ALT, alanine aminotransferase.

^*^Active cancer was defined as malignancy confirmed with histological or cytological evidence and having one or more of the following features: diagnosis of cancer within the 6 months; progressive disease; or cancer treatment during the 6 months.

### Characteristics of stroke and 3-month mortality

**Figure 1:**
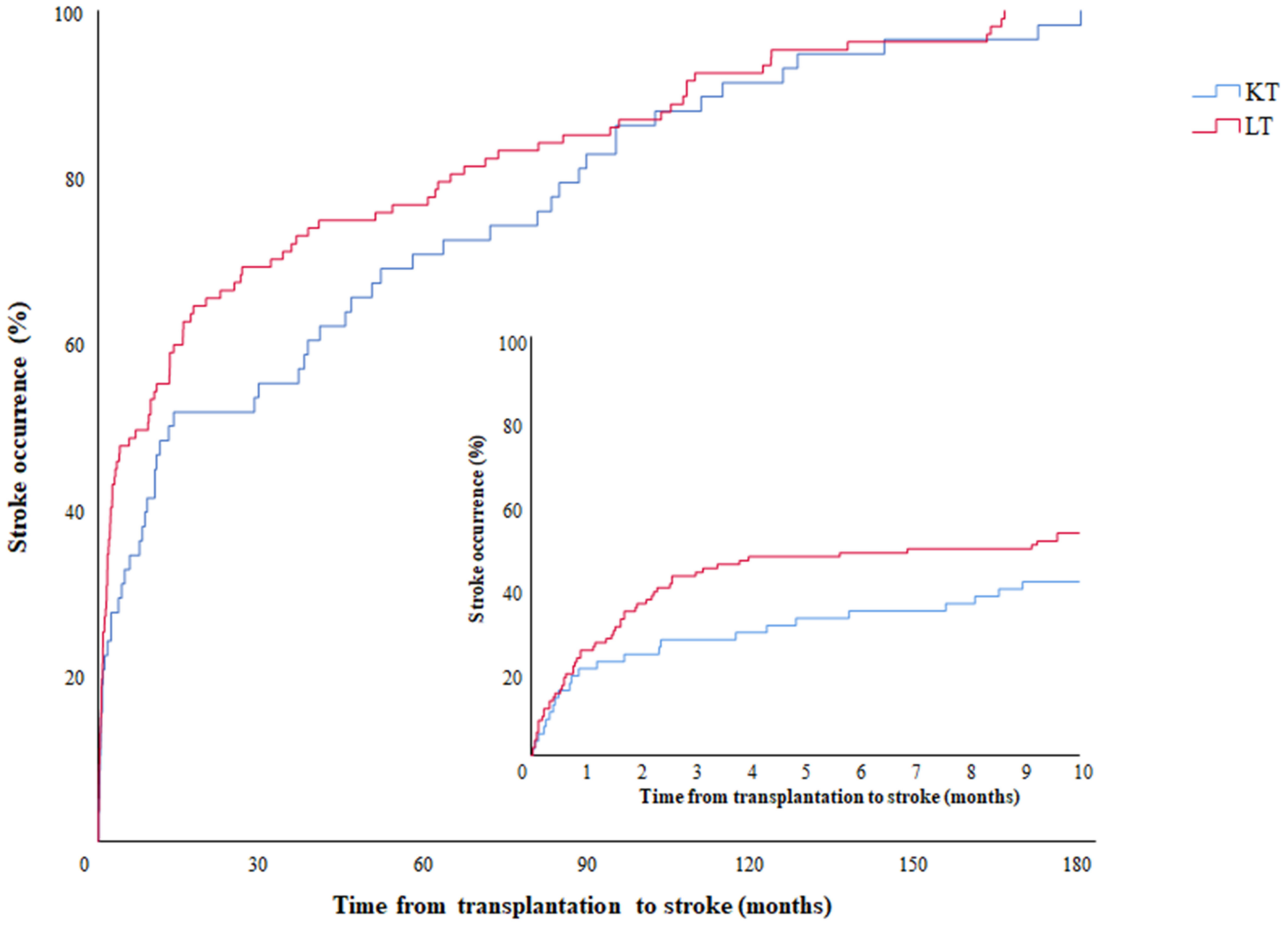
Occurrence of stroke events according to time from transplantation. Kaplan-Meier graph showing the occurrence of stroke according to time. The red and blue lines represent the LT and KT groups, respectively. The embedded graph describes the same data on a more detailed time scale. LT, liver transplantation; KT, kidney transplantation.

Table 2 shows the features of stroke in the LT and KT groups. Among the 105 recipients of the LT group, two of them experienced both ischemic and hemorrhagic strokes simultaneously and counted twice. The occurrence of strokes tends to happen earlier in the LT group than the KT group, as illustrated in Figure 1, but the difference did not reach statistical significance (median 274.0 days vs. 401.0 days; *p* = 0.11). On the other hand, the LT group had a higher incidence of postoperative 3-month stroke (43.9 vs. 27.6%, *p* = 0.04), In-hospital stroke (57.9 vs. 39.7%, *p* = 0.03), and hemorrhagic stroke (25.2 vs. 8.6%, *p* = 0.01) compared do the KT group.

**Table 2:** Characteristics of post-transplantation stroke and 3-month mortality rates in the participants.

**Characteristics**	LT group (*n* = 107)^*^	**KT group (*n* = 58)**	***p*-value**
Transplantation to stroke duration (day)	274.0 (27.0–1524.0)	401.0 (65.3–2434.0)	0.11
Postoperative 3-month stroke	47 (43.9)	16 (27.6)	0.04
In-hospital stroke	62 (57.9)	23 (39.7)	0.03
**Stroke**			0.01
Hemorrhagic stroke	27 (25.2)	5 (8.6)	
Ischemic stroke	80 (74.8)	53 (91.4)	
**TOAST**			0.19
Large artery atherosclerosis	9 (11.3)	9 (17.0)	
Small vessel occlusion	25 (31.3)	13 (24.5)	
Cardioembolic	10 (12.5)	13 (24.5)	
Other determined	6 (7.5)	1 (1.9)	
Undetermined	30 (37.5)	17 (32.1)	
Multiple lesions	34 (42.5)	26 (49.1)	0.46
Cortical lesion	40 (50.0)	27 (50.9)	0.92
Hemorrhagic transformation^†^	6 (15.4)	7 (23.3)	0.40
**Fazekas score**			0.46
Grade 0	38 (35.5)	28 (48.3)	
Grade 1	48 (44.9)	20 (34.5)	
Grade 2	17 (15.9)	8 (13.8)	
Grade 3	4 (3.7)	2 (3.4)	
Microbleeds^‡^	21 (26.6)	11 (26.2)	0.96
Endovascular treatment	1 (0.9)	0 (0.0)	>0.99
Intravenous tPA injection	1 (0.9)	1 (1.7)	>0.99
**Discharge antithrombotics**			0.02
Mono antiplatelet therapy	26 (24.3)	11 (19.0)	
Dual antiplatelet therapy	21 (19.6)	22 (37.9)	
Anticoagulation therapy	12 (11.2)	10 (17.2)	
None	48 (44.9)	15 (25.9)	
3-month mortality	11 (10.3)	6 (10.3)	>0.99

Numbers are presented as the median (interquartile range) or number (percentage).

LT, liver transplantation; KT, kidney transplantation; TOAST, Trial of Org 10,172 in Acute Stroke Treatment; tPA, tissue plasminogen activator.

^*^Two patients presented both ischemic and hemorrhagic stroke and were included in both groups.

^†^Patients with ischemic stroke and available follow-imaging within 2 weeks were analyzed (LT, *n* = 39; KT, *n* = 30).

^‡^Patients with available gradient echo imaging were analyzed (LT, *n* = 79; KT, *n* = 42).

The distribution of TOAST classification did not show a significant difference between LT and KT groups. In both the groups, undetermined etiology was the most common cause of stroke. More specifically, in the LT group, the most frequent cause of the stroke was undetermined etiology (*n* = 30, 37.5%), which consisted of in the order of negative etiology (*n* = 25), incomplete evaluation (*n* = 4), and 2 or more etiologies (*n* = 1). The second most common etiology in the LT group was SVO (*n* = 25, 31.3%), followed by cardioembolism (*n* = 10, 12.5%), LAA (*n* = 9, 11.3%), and other determined etiology (*n* = 6, 7.5%). In six other determined etiology patients, air embolism (*n* = 2), cervical artery dissection (*n* = 1), cancer-related stroke (*n* = 2), and coronary artery angiography-related stoke (*n* = 1) were the causes of stroke.

In the KT group, the undetermined etiology (*n* = 17, 32.1%) was the most frequent etiology as well, followed by SVO (*n* = 13, 24.5%) and cardioembolism (*n* = 13, 24.5%). The undetermined etiology comprised of 15 cases with negative etiology, 1 with 2 or more etiologies, and 1 with incomplete evaluation. One (1.9%) patient with other determined etiology was diagnosed with Moyamoya disease. Further evaluation of imaging features such as multiplicity, cortical involvement, and hemorrhagic transformation did not differ between the two groups. The presence of microbleeds and Fazekas score were also similar between the two groups. At discharge, a higher proportion of LT patients (44.9 vs. 25.9%, *p* = 0.02) did not receive antithrombotic agents.

The all-cause 3-month mortality was approximately 10% in the both groups (KT, 10.3%; LT, 10.3%; *p* > 0.99). Results of the multivariate logistic regression analysis (Table 3) showed in-hospital stroke (odds ratio [OR], 4.17; 95% confidence interval [CI], 1.07–16.30) and elevated C-reactive protein (OR, 1.10; 95% CI, 1.01–1.20) levels were related to all-cause mortality. After conducting a subgroup analysis, we found that in the LT group, C-reactive protein level was independently associated with an increased risk of 3-month mortality after the multivariable analysis, while no significant factors were identified in the KT group (Supplementary Tables 1, 2).

**Table 3:** Regression analysis for 3-month mortality in participants.

**Univariable**			**Multivariable**	
**Factor**	**OR (95% CI)**	***p*** **-value**	**OR (95% CI)**	***p*** **-value**
Liver transplantation	0.99 (0.35–2.84)	0.99		
Age	0.99 (0.95–1.04)	0.77		
Male sex	0.83 (0.27–2.51)	0.74		
Previous stroke	0.13 (0.20–0.98)	0.05	0.16 (0.02–1.32)	0.09
Diabetes mellitus	0.80 (0.29–2.22)	0.67		
Hypertension	0.67 (0.24–1.83)	0.43		
Atrial fibrillation	0.65 (0.08–5.29)	0.69		
Active cancer	2.67 (0.85–8.38)	0.09		
In-hospital stroke	5.06 (1.40–18.35)	0.01	4.17 (1.07–16.30)	0.04
Hemorrhagic stroke	0.53 (0.11–2.42)	0.41		
C-reactive protein	1.10 (1.02–1.19)	0.02	1.10 (1.01–1.20)	0.03
Creatinine	0.97 (0.68–1.39)	0.87		
AST	1.00 (1.00–1.01)	0.13		
ALT	1.00 (0.99–1.01)	0.92		

AST, aspartate aminotransferase; ALT, alanine aminotransferase; OR, odds ratio; CI, confidence interval.

## Discussion

In this study, we investigated the characteristics of post-transplantation stroke in the KT and LT recipients. Although patients in the KT group had more vascular risk factors than those in the LT group, undetermined etiology was the most common cause of stroke in both groups. In the LT group, in-hospital stroke, peri-operative stroke, and hemorrhagic stroke were more frequently observed than in the KT group. Mortality at 3 months was similar between the two groups, but was higher than that in the general stroke population in Korea (22).

Previous studies have reported the incidence of post-transplantation stroke, varying from 1.1 to 8.0% (5, 6, 13, 23, 24) in KT recipients and 1.8 to 4% (8–10) in LT recipients. Our transplantation recipients presented with a relatively lower incidence of 1.3 and 1.8%, respectively. This was probably due to the correct management of vascular risk factors after transplantation, as well as the use of newer immunosuppressants, which are known to have fewer side effects (25–27). The relatively low proportion of LAA etiology in our KT recipients despite the prevalence of cardiovascular risk factors would be attributable to the same reasons. Conversely, LT recipients showed a higher rate of hemorrhagic stroke in accordance with increased bleeding tendency reflected in longer coagulation time and lower platelet count.

Although the frequency order of etiology in ischemic stroke showed a difference, it did not reach statistical significance. In both groups, undetermined etiology was the most common cause of stroke. LT recipients were more likely to suffer a stroke during the early postoperative period, when they were prone to inflammatory status and hospitalization, mostly due to infection.

Among the 63 postoperative 3-month stroke, 19 (40%) were classified as undetermined cause ischemic strokes. They showed higher median level of CRP and lower median platelet count compared to other ischemic stroke. (Data not shown) Additionally, the lesions were multifocal in most of patient (*n* = 17), suggesting a potential role of inflammatory and thorombogenic condition in the development of stroke in this group of patients. Inflammation is known to be related to activation of the coagulation system and associated with increased embolic stroke risk (28). More than half of all ischemic strokes occurred during hospitalization, thus, the post-operative status may at least partially explain the high proportion of ischemic stroke classified as undetermined etiology in LT recipients.

On the other hand, KT recipients were more likely to suffer a stroke during everyday life, and the onset of stroke was relatively delayed compared to that in LT recipients. Considering these different profiles, additional factors other than post-operative condition would affect the stroke occurrence in KT recipients. Previous studies have reported that CKD patients had a high rate of atrial fibrillation (29) and the most common cause of ischemic stroke among the CKD patients was cardioembolism followed by undetermined etiology, accordingly (30). In addition, studies of KT recipients found that atrial fibrillation was an important predictor of stroke (6, 31), and the incidence of new-onset atrial fibrillation was relatively frequent after KT (31, 32). Our results are not contradictory to the previous literature, in that undetermined etiology and cardioembolism were the two most frequent etiologies among CKD patients. However, the difference may have resulted from unrevealed embolic sources such as paroxysmal than persistent atrial fibrillation due to the younger age of KT recipients than that of CKD patients (33, 34), combined with the unavailability of prolonged cardiac monitoring in all patients, thereby increasing the proportion of stroke of undetermined etiologies.

We found that SVO was the second most frequent etiology in both LT and KT recipients. It is well-known that SVO is common in those with renal impairment (35). The anatomical similarity of possessing strain vessels, which are perforators and glomerular afferent arterioles, may explain the high incidence of SVO in KT recipients (36). Interestingly, LT recipients were also prone to SVO. Previous studies evaluating brain MRI findings in liver cirrhosis and fatty liver patients found increased white matter changes (37–40) or a higher proportion of lacunar infarction (41). The exact mechanism is still unknown, but cerebral edema related to hepatic encephalopathy (37), decreased cerebrovascular reactivity, and subclinical inflammation leading to endothelial dysfunction are all considered to play a role in increased white matter lesions and lacunar infarction (39). Although the patients received a new graft, endothelial dysfunction in small perforators in the brain persisted and may have affected SVO occurrence in LT recipients.

We found that all-cause mortality at 3 months was similar in both groups, but higher than in the general Korean stroke population (22). In particular, in-hospital stroke and elevated C-reactive protein were associated with increased mortality. The fragile conditions related to hospitalization and increased inflammatory markers such as infections could contribute to the high mortality. Although the incidence of stroke is not frequent in LT and KT recipients, preventing stroke is still important because the recipients are generally young, so the effect of disability and functional graft loss caused by stroke would be substantial both personally and socially.

This study has some limitations which should be noted. First, the single-center design may have introduced potential bias. However, patient data were collected from a large volume center where more than 400 LTs and 400 KTs are performed annually. Second, despite their clinical significance in determining outcomes, important measures of stroke severity such as the National Institutes of Health Stroke Scale score and the Glasgow Coma Scale score were not available in this study. As a result, the factors identified for 3-month mortality may have limitations and potentially suffer from bias. Third, comprehensive evaluations of possible embolic sources were not feasible in this study, especially for in-hospital stroke patients. Nevertheless, all patients underwent routine 12-lead EKG as part of the original TOAST classification criteria, and therefore, the classification is not affected by the lack of more advanced exams. Lastly, some patients may have been admitted to other centers, and thus were not included. However, most patients would have been admitted to this hospital since the transplantation center coordinator takes calls from the patients and arranges for them to be admitted.

Overall, in this study we compared the characteristics and etiologies of post-transplantation stroke in LT and KT recipients for the first time, in a single but large-volume center. Although undetermined etiology was the most common cause of ischemic stroke in both groups, hemorrhagic, perioperative, and in-hospital strokes were more frequent in the LT group. Mortality rates at 3 months were similar but higher than that in the general population in both groups, which indicates the need for close medical observation in such patients. For a better understanding of stroke mechanisms and effective prevention in this group of patients, further investigations with prospective design are required.

## Data availability statement

The raw data supporting the conclusions of this article will be made available by the authors, without undue reservation.

## Ethics statement

The studies involving human participants were reviewed and approved by Institutional Review Board (IRB) of Asan Medical Center, Korea (IRB approval number: 2021-0475). Written informed consent for participation was not required for this study in accordance with the national legislation and the institutional requirements.

## Author contributions

HK acquired, analyzed, and interpreted the data and wrote the manuscript. SS and CB interpreted the data revised the manuscript. BK designed and conceptualized the study, interpreted the data, and revised the manuscript. JC, D-WK, SK, and JK revised the manuscript. All authors contributed to the article and approved the submitted version.
