# Supplementary material for: Characteristics of stroke after liver and kidney transplantation
Source: Front Neurol. 2023 Mar 22;14:1123518. doi: 10.3389/fneur.2023.1123518 (PMC10073414; doi:10.3389/fneur.2023.1123518)
Supplement: Supplementary file 1 [file Table_1.DOCX]

**Supplement Table 1.** Regression analysis for 3-month mortality in the KT group.

| Univariable | | | Multivariable | |
| --- | --- | --- | --- | --- |
| Factor | **OR (95% CI)** | ***p*-value** | **OR (95% CI)** | ***p*-value** |
| Age | 0.99 (0.92 - 1.05) | 0.68 |  |  |
| Male sex* | - | - |  |  |
| Previous stroke | 0.27 (0.03 - 2.50) | 0.25 |  |  |
| Diabetes mellitus | 0.20 (0.03 - 1.23) | 0.08 |  |  |
| Hypertension | 0.67 (0.24–1.83) | 0.43 |  |  |
| Atrial fibrillation | 1.53 (0.15 - 15.44) | 0.72 |  |  |
| Active cancer† | - | - |  |  |
| In-hospital stroke | 3.47 (0.58 - 20.78) | 0.17 |  |  |
| Hemorrhagic stroke‡ | - | - |  |  |
| C-reactive protein | 1.08 (0.95 - 1.22) | 0.26 |  |  |
| Creatinine | 1.38 (0.79 - 2.42) | 0.26 |  |  |
| AST | 1.01 (0.99 - 1.03) | 0.35 |  |  |
| ALT | 1.00 (0.97 - 1.04) | 0.96 |  |  |

Abbreviations: KT, Kidney transplantation; CRP, C-reactive protein; ALT, alanine aminotransferase; AST, aspartate aminotransferase; CI, confidence interval; OR, odds ratio

*, †, ‡Binary logistic regression analysis could not be performed for these variables since the event was not present in one of the categories.

**Supplement Table 2.** Regression analysis for 3-month mortality in the LT group.

| Univariable | | | Multivariable | |
| --- | --- | --- | --- | --- |
| Factor | **OR (95% CI)** | ***p*-value** | **OR (95% CI)** | ***p*-value** |
| Age | 1.00 (0.94 - 1.07) | 0.99 |  |  |
| Male sex | 0.36 (0.10 - 1.28) | 0.11 |  |  |
| Previous stroke* | - | - |  |  |
| Diabetes mellitus | 1.67 (0.47 - 5.88) | 0.43 |  |  |
| Hypertension | 0.57 (0.16 - 2.10) | 0.40 |  |  |
| Atrial fibrillation† | - | - |  |  |
| Active cancer | 3.87 (1.06 - 14.17) | 0.04 | 3.81 (0.91-16.01) | 0.07 |
| In-hospital stroke | 8.46 (1.04 - 68.72) | 0.05 | 11.16 (0.70-178.78) | 0.09 |
| Hemorrhagic stroke‡ | - | - |  |  |
| C-reactive protein | 1.13 (1.01 - 1.27) | 0.04 | 1.18 (1.05-1.34) | 0.01 |
| Creatinine | 0.70 (0.29 - 1.70) | 0.43 |  |  |
| AST | 1.00 (1.00 - 1.01) | 0.16 |  |  |
| ALT | 1.00 (0.99 - 1.01) | 0.89 |  |  |

Abbreviations: LT, Liver transplantation; CRP, C-reactive protein; ALT, alanine aminotransferase; AST, aspartate aminotransferase; CI, confidence interval; OR, odds ratio

*, †, ‡Binary logistic regression analysis could not be performed for these variables since the event was not present in one of the categories.
